# Endocrine-disrupting properties of heavy metal exposure contribute to the intergenerational effects on learning and memory

**DOI:** 10.3389/fendo.2026.1761556

**Published:** 2026-03-26

**Authors:** Glaecir Roseni Mundstock Dias, Helena D. Zomer, Nyam F. Silva, Jones B. Graceli

**Affiliations:** 1 Programa de Pós-Graduação em Medicina (Endocrinologia), Faculdade de Medicina, Universidade Federal do Rio de Janeiro, Rio de Janeiro, RJ, Brazil; 2 Centro de Pesquisa em Medicina de Precisão, Instituto de Biofísica Carlos Chagas Filho, Universidade Federal do Rio de Janeiro, Rio de Janeiro, RJ, Brazil; 3 Department of Physiological Sciences, College of Veterinary Medicine, University of Florida, Gainesville, FL, United States; 4 Departamento de Morfologia, Universidade Federal do Espírito Santo, Vitória, ES, Brazil; 5 Animal Science, School of Agricultural Sciences, Southern Illinois University, Carbondale, IL, United States

**Keywords:** behavior, cadmium, endocrine-disrupting chemicals, heavy metals, intergenerational, lead, methylmercury

## Abstract

Heavy metals are increasingly recognized as endocrine-disrupting chemicals (EDCs) capable of perturbing neurodevelopment and cognition across multiple generations. Among these, methylmercury (MeHg), lead (Pb), and cadmium (Cd) remain global public health concerns due to their environmental persistence, bioaccumulation in food and water sources, and widespread human exposure. Evidence from epidemiological and experimental studies demonstrates that these metals can interfere with hormonal signaling, neurogenesis, synaptic organization, neuroendocrine regulation and epigenetic programming—processes that are essential for learning and memory formation during critical developmental windows. Collectively, current evidence supports the view that MeHg, Pb, and Cd exposure act as potent neuroendocrine disruptors capable of producing intergenerational consequences on learning and memory. In this review, we highlight the recent findings of the effects of prenatal exposure to the three heavy metals (i.e., MeHg, Pg and Cd) on developing learning and memory.

## 1 Introduction

Metal pollution, particularly from heavy metals, is a significant global public health problem (1–3). Heavy metals do not have a biological function, being toxic to biological systems at any concentration. Changes in land use, including agriculture, mining, urbanization and deforestation have altered the natural biogeochemical cycles of metals and leading to their remobilization. Due to their widespread presence in the biosphere, certain metals, such as mercury (Hg), lead (Pb) and cadmium (Cd), have been classified as global pollutants (UN 4, 5). Heavy metals were categorized as endocrine-disrupting chemicals (EDCs) in recent decades due to their toxicity to the endocrine system (6–11).

Metallic mercury (Hg^0^) is vaporized from the Earth’s surface naturally or by anthropogenic emissions, being oxidized in the atmosphere to divalent inorganic mercury (Hg²^+^), which deposits in soils and waters through dry and wet deposition. The Hg²^+^, which is primarily associated with suspended particulate matter and is transported through aquatic systems, is deposited in sedimentary aquatic environments, where it is subjected to optimal biogeochemical conditions for methylation (12). The resulting methylmercury (MeHg) is incorporated into organisms along the food chain, bioaccumulating and biomagnifying until it reaches humans, mainly through the consumption of contaminated fish, shellfish, marine mammals, and other foods (4).

Stable organic compounds such as tetraethyl lead (Pb) and tetramethyl lead can be produced from Pb, which occurs naturally in the Earth’s crust, particularly in regions enriched with phosphatic rocks and marine sediments. Beginning in the 1920s, these organolead compounds were added to gasoline, transforming lead into a pervasive pollutant that was dispersed worldwide through atmospheric transport, reaching ecosystems far from natural or anthropogenic sources. Although the United States completed its ban on leaded gasoline in 1996 and the global phase-out was finalized in 2021, its use has left a profound and lasting environmental impact (13). The capacity of biota to absorb Pb directly from atmospheric deposition, or indirectly via transfer from soil and water into plants and subsequently animals, contributes to its bioaccumulation and long-term ecological and health risks (14).

Cadmium (Cd) was first produced industrially in the 20th century as a byproduct of the zinc industry. Originally utilized for electroplating, pigments, and plastic stabilizers, it is now utilized in the manufacturing of nickel-cadmium batteries and is a crucial component of many contemporary technologies with uses in the energy generation, electronics, communications, and aerospace industries. Cd’s long half-life (20–40 years) is its primary metabolic feature, leading to a lifetime of nearly irreversible accumulation in the body. It mostly binds to metallothioneins and accumulates in the liver and kidneys (2, 15).

Experimental and epidemiological studies have demonstrated that MeHg, Pb, and Cd are strong neurotoxins that cause a range of neurological, behavioral, motor, and visual impairments in both laboratory animals and humans (16–19). Their neurotoxic effects involve multiple mechanisms, including the production of reactive oxygen species (ROS), disruption of the antioxidant balance, apoptosis, and impairment of cellular signaling pathways critical for neuronal growth, differentiation, and synaptic plasticity. They also interfere with essential metal homeostasis; for example, Pb disrupts calcium signaling pathways, while Hg interferes with enzymes requiring zinc (Zn) or selenium (Se). Additional mechanisms include damage to the blood-brain barrier, disruption of neurotransmitter systems, alterations in the microbiome, and epigenetic modifications (20–23).

EDCs properties of heavy metals were identified in several models of exposure and demonstrated in epidemiological studies. MeHg, Pb, and Cd accumulate in the thyroid gland and disrupt thyroid hormone production, leading to prolonged thyrotoxicity and hormonal imbalances (11, 24–26). In addition to the effects on thyroid gland, heavy metals are involved in reproductive and metabolic disorders, such as diabetes and obesity (15, 27–29). EDCs mechanisms of MeHg, Pb, and Cd are carried out mainly via hormone receptors, besides changes in enzymatic pathways for biosynthesis and metabolism of hormones, but also in numerous other mechanisms, such as oxidative stress (30). As a EDCs, MeHg, Pb, and Cd exposure is associated to display nonmonotonic dose-response (NMDR) relationships, including inverted U-shaped curves, where biological effects do not increase linearly with dose but instead peak at intermediate concentrations and decline at both lower and higher doses. These patterns contrast with traditional toxicology assumptions — “the dose makes the poison” — and reflect fundamental endocrine system dynamics, such as receptor binding affinities, feedback regulation, and differential cellular responses to low versus high exposures (31, 32).

The nervous system and the endocrine system are inextricably linked, and they are both actively involved in the maintenance of homeostasis through bidirectional interaction. Hormones can alter behavior, and behavior can occasionally influence hormone concentrations (33). Endocrine dysfunctions can lead to various neurological manifestations, demonstrating the complex interplay between these systems. Exposure to EDCs can induce behavioral modifications in both directly exposed individuals and subsequent generations, resulting in inter- and transgenerational effects that are attributed to the modification of epigenetic regulation (32).

Changes in the brain at the synaptic and cellular level are shaped by processes such as synaptic plasticity, neurotransmitter dynamics, and hormonal responses, which together form the neurobiological basis of learning and memory. The encoding, storage, and retrieval of information are all dependent on these changes, with various brain regions fulfilling distinct functions. The initial processing of information, which frequently involves synaptic changes, is referred to as encoding. Storage is the process of retaining information over time, which involves the consolidation of cellular and system components. Retrieval is the process of accessing stored information (34). Hormones important to stress, reproductive status, and motivational status appear to create conditions that favor learning certain aspects of a situation more than others. In particular, hormones differentially regulate the balance among memory systems, which leads to an animal employing one or more strategies to learn a task and subsequently retaining a more considerable amount of information about one aspect of the task (35–38).

In this review, we examine the literature on endocrine-disrupting properties of heavy metals (MeHg, Pb, and Cd) and their potential links to intergenerational behavioral changes, i.e., effects observed in the progeny of various exposure models, depending on the exposure period, dosage, and behaviors assessed, with particular emphasis on learning and memory. PubMed, ScienceDirect, and Google Scholar platforms were employed to conduct a literature search from January to December 2025. Heavy metals, methylmercury, lead, cadmium, behavior, learning, memory, and endocrine disruptors were implemented as keyword strategies. The redundancy of information was examined in papers written in English from the year 2000 onward, and they were subsequently included or excluded. Reference tracking was employed to acquire additional pertinent publications.

## 2 Heavy metals and intergenerational consequences

### 2.1 MeHg EDCs properties

MeHg interferes with the normal functioning of hormones in living organisms. The hypothalamic-pituitary-gonadal (HPG) axis is among the endocrine systems that have been demonstrated to be impacted by it, resulting in reproductive issues in both laboratory and wild animals (39). Studies have indicated that MeHg affects the HPG axis (testes and ovaries) (40, 41), leading to abnormal production and release of sex (testosterone and estradiol) and gonadotropin (LH- Luteinizing hormone and FSH- Follicle-stimulating hormone), and behaviors related to reproduction, such as foraging and parental care, can all be impacted by MeHg exposure (41–43).

MeHg (as well as other environmental contaminants, including pesticides, polychlorinated biphenyls, dioxins, phthalates, bisphenols, and other heavy metals), have been identified as contributing factors to the heightened prevalence of thyroid disease and thyroid cancer in recent decades (11, 44). MeHg compounds are classified as possibly carcinogenic to humans (Group 2B) (45). The tumorigenic effects of MeHg were investigated in Nthy-ori-3–1 cells (immortalized, non-tumorigenic normal thyroid cells). Following a 24-hour exposure to MeHg at 2.5 and 5 µM, the cell viability decreased, resulting in a decrease in the sub-G0 phase cell population. Conversely, MeHg at a lower concentration of 0.1 µM increased the cell viability, resulting in a rise in the G2/M phase cell population. The prolonged exposure of the cells to 0.1 µM MeHg for up to 18 days resulted in an increase in thyroid cell proliferation by activating the ERK-mediated pro-oncogenic signal transduction pathway (10).

MeHg can also disrupt the function of adrenal glands, affecting the production of catecholamines such as noradrenaline and adrenaline. There is evidence from rodent studies that MeHg disrupts the adrenal medulla’s capacity to synthesize these hormones, resulting in altered levels in the bloodstream. This leads to downstream effects on various bodily functions, particularly those regulated by the sympathetic nervous system (46). A recent retrospective autopsy study reported that the proportion of individuals with mercury in the adrenal medulla increases steadily after the age of 20, reaching 90% or more in those over 80 years. This accumulation suggests that mercury may alter catecholamine metabolism in the adrenal gland, potentially contributing to the age-associated decrease in adrenaline secretion and the rise in plasma noradrenaline. Such changes could, in turn, play a role in the sympathetic overactivity linked to metabolic syndrome and primary hypertension (47).

The relationship between obesity and MeHg exposure is intricate and far from fully comprehended. Studies have demonstrated both positive and negative associations, contingent upon the population studied and the type and time of mercury exposure. While some studies suggest a negative correlation between blood Hg levels and obesity in adults (48), others, particularly in Asian populations, demonstrate a positive correlation (49). In 3T3-L1 cells, MeHg has also been demonstrated to have the potential to affect adipogenesis and lipid metabolism. Lipid droplet formation and adipokine expression are stimulated by 0.5 µM MeHg exposure, particularly in the latter stages of adipocyte differentiation. This process involves increased triglyceride (TG) content, larger numbers of lipid droplets, and upregulation of adipogenic genes, including peroxisome proliferator-activated receptor γ (PPARγ), adiponectin, and fatty acid-binding protein (50).

In addition, a relationship between MeHg exposure and diabetes is not entirely comprehended; however, certain studies indicate an inverse correlation, which implies that greater MeHg levels may be associated with a reduced risk of developing diabetes (51). Other studies demonstrate no significant associations or mixed results, and some even suggest a positive correlation (52, 53). Additional research is required to elucidate the impact of MeHg on the risk of diabetes, with a particular emphasis on the potential modification of the relationship by factors such as selenium and omega-3 fatty acid intake (51). It has been demonstrated that pancreatic β-cells are susceptible to MeHg toxicity due to oxidative stress damage, which results in apoptosis and dysfunction (54, 55).

In mice treated with MeHg chloride (2 mg/kg) for more than 2 consecutive weeks, blood glucose levels increased, and plasma insulin secretions decreased. An increase in TUNEL-positive cells were observed in the islets (apoptosis), which was accompanied by a decrease in the expression of the mRNA of anti-apoptotic genes (Bcl-2, Mcl-1, and Mdm-2), an increase in the apoptotic (p53, caspase-3, and caspase-7) and a decrease in the genes related to antioxidant defense, including Nrf2, GPx, and NQO1 (54). The viability of RIN-m5F cells derived from pancreatic β-cells and insulin secretion was reduced by MeHg (1-4 μM). It was observed a concurrent increase in mitochondrial-dependent apoptotic events, such as cytochrome c release, annexin V-Cy3 binding, caspase-3 activity, and caspase-3/-7/-9 activation, as well as a decrease in mitochondrial membrane potential and an increase in the proapoptotic (Bax, Bak, p53)/antiapoptotic (Bcl-2) mRNA ratio. The protein expression of endoplasmic reticulum (ER) stress-related signaling molecules, such as C/EBP homologous protein (CHOP), X-box binding protein (XBP-1), and caspase-12, was also induced by the exposure of RIN-m5F cells to MeHg (2 μM) (55).

**Figure 1:**
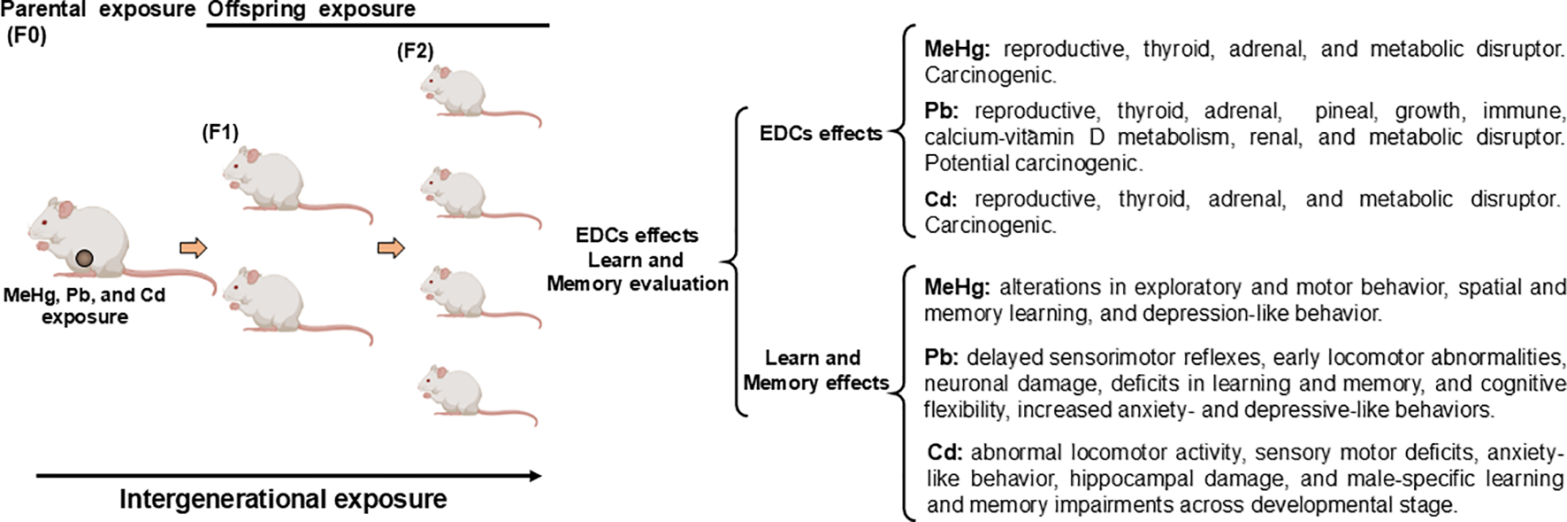
Intergenerational effects of methylmercury (MeHg), lead (Pb) and cadmium (Cd) exposure as an endocrine-disrupting chemical (EDCs) in learn and memory. Embryonic exposure involves exposure of the F0 generation in female pregnancy, the F1 generation embryo (marked in brown in the diagram), and the germline of the F2 generation. This intergenerational exposure indicates that the phenotypes of the F0-F2 generations may be due to direct exposure to MeHg, Pb, and Cd.

Thus, MeHg is a potent EDCs contaminant that interferes with multiple hormonal systems, including the endocrine, nervous and reproductive system. MeHg is also linked to metabolic homeostasis abnormalities, such as positive and negative associations between MeHg burden and obesity, while *in vitro* evidence shows that MeHg enhances adipogenesis, lipid accumulation, and adipokine expression. Although epidemiological findings on MeHg and diabetes risk remain inconsistent, mechanistic studies consistently reveal that pancreatic β-cells are highly susceptible to MeHg-induced oxidative stress, mitochondrial and ER-mediated apoptosis, and impaired insulin secretion. Collectively, these findings highlight MeHg as broad-spectrum EDCs with significant implications for reproductive, thyroid, adrenal, and metabolic health (**Figure 1**).

### 2.2 MeHg perinatal exposure and behavior effects

Perinatal MeHg exposure has been associated with alterations in exploratory and motor behavior, spatial learning, memory, and depression-like behavior (**Table 1**). Giménez-Llort et al. (56), Daré et al. (59) demonstrated that prenatal and early postnatal exposure to MeHg affected motor behavior and spatial learning (**Figure 1**). Brain Hg concentrations were comparable between male and female rats at postnatal day (PND) 14 (≈300 ng total Hg/g w/w) and PND 21 (≈100 ng total Hg/g w/w). At PND 14, motor activity was significantly increased in both sexes, however, sex-dependent differences emerged at PND 21 in response to dopaminergic D2 agonist U913456A (0.1 mg/kg). Male rats displayed a marked reduction in motor activity following postsynaptic dopamine D2 receptor stimulation, suggesting that the early exposure to MeHg disrupts dopaminergic function. The authors further proposed that the endocrine-disrupting properties of MeHg on adrenal and gonadal functions may contribute to altered dopamine neurotransmission (56). No changes were detected in motor coordination (Rotarod Test), but male offspring exhibited increased motor activity in response to apomorphine at PND 20, while spatial learning deficits became evident at PND 60 (59).

**Table 1:** MeHg perinatal exposure schemes.

Species	Dose of MeHg used and route of administration	Exposure period	Time of behavioral analyses in the offspring*	Reference
Sprague-Dawley rats	0.5 mg MeHg/kg/day, oral route by drinking water	GD 7 - PND 7	PND 14 PND 21	Giménez-Llort et al. (56)
Sprague-Dawley rats	8 mg MeHg/kg, oral route by intragastric intubation	GD 8	PND 60 (No sex specification)	Baraldi et al. (57)
Wistar rats	5 ppm MeHg, oral route by diet	From 8 weeks to conception, gestation and lactation (until PND 30) (only female rats) Young rats continued at the same diet for 60 days	PND 35 PND 42	Sakamoto et al. (58)
Sprague-Dawley rats	0.5 mg MeHg/kg/day, oral route by drinking water	GD 7 - PND 7	PND 20 PND 21 PND 60	Daré et al. (59)
C57BL/6 mice	4, 6, or 8 ppm MeHg/kg, oral route by drinking water	GD 2 - PND 21	6 weeks (PND 42) 12 weeks (PND 84)	Goulet et al. (60)
Long-Evans rats	0.5, or 6.5 ppm MeHg, oral route by drinking water, corresponding to 40-50 or 500-700 µg/kg/day	4 weeks before mating until PND 16 (only female rats)	1.7 years old 2.3 years old	Newland et al. (61)
Long-Evans rats	0.5 ppm MeHg, oral route by drinking water	4 weeks before mating until PND 16 (only female rats)	PND 60	Roegge et al. (62)
Female B6C3F1/ HSD mice Male CBA/J HSD mice Offspring trihybrid B6C3F1/HSD CBA/J HSD	1 ppm, or 3 ppm MeHg in a5 nM sodiumcarbonatedrinking solution, oral route by drinking water	4 weeks before mating (female mouse) During mating (male and female mouse) GD 0 until PND13 (perinatal exposure) Lifetime exposure of the offspring	5 months 15 months 22 months 26 months	Weiss et al. (63)
Sprague-Dawley rats	0.5 or 1.0 mg MeHg/kg/day, oral route by intragastric intubation	4 weeks before mating (female rats) until GD 20	PNDs 17, 21, 22, and 35- 38	Beyrouty et al. (64)
Sprague-Dawley rats	8 mg MeHg/kg, oral route by intragastric intubation	GD 8 or 15	PND 40	Carratù et al. (65)
C57BL/6/Bkl mice	0.5 mg MeHg/kg/day, oral route by drinking water	GD 7 - PND 7	5 - 15 and 26 - 36 weeks of age	Onishchenko et al. (66)
C57Bl/6J mice	0.01 mg MeHg/kg, oral route by contaminated food	GD 8 - GD 18	PND 60 (motor activity) PND 180 (memory assessment)	Montgomery et al. (67)
C57BL/6Cr mice	5 ppm MeHg, oral route by diet	From 4 weeks to conception, gestation and lactation (until PND 30) (only female rats)	Preweaning developmental assessment (both sex): PNDs 4, 7, 10, 12, 14 and 16. Postnatal behavioral tests (male sex): 8 weeks of age	Sugawara et al. (68)
Sprague-Dawley rats	4 mg MeHg/kg and 8 mg MeHg/kg, oral route by intragastric intubation	GD 15	PND 90	Ferraro et al. (69)
Wistar rats	0.5 mg/kg/day or 2.0 mg/kg/day (estimated), oral route by drinking water	GD 7 - PND 21	13 - 20 weeks of age	Gralewicz et al. (70)
Wistar rats	8 mg MeHg/kg, oral route by intragastric intubation	GD 15	PND 60 (male and female analyzed together)	Maia et al. (71)
Wistar rats	8 mg MeHg/kg, oral route by intragastric intubation	GD 15	PND 60	Maia et al. (72)

MeHg, methylmercury; GD, gestational days; PND, postnatal days. *Male and female separated or as described in the line.

While behavioral studies did not reveal significant changes in female offspring exposed to MeHg during development, male mice exhibited impairments in learning ability and motivational behavior, along with a persistent predisposition to depressive-like behavior (66, 70). Onishchenko et al. (66) further demonstrated long-lasting effects in both young and adult animals, reporting a predisposition to depressive-like behavior in males exposed to MeHg in early life. At PND 8, pregnant mice exposed to MeHg had whole-brain Hg concentrations of 2.60 ± 0.19 µg/g wet weight, while their pups’ showed concentrations of 0.93 ± 0.02 µg/g.; by 4 weeks of age, brain mercury levels in the offspring had declined sharply to 0.045 ± 0.003 µg/g. Interestingly, both male and female MeHg-exposed mice were less active than controls during the dark period, suggesting disturbance of the sleep-wake cycle.

Carratù et al. (65) demonstrated that prenatal MeHg exposure induces subtle behavioral abnormalities in male rat offspring, even at dose levels below those causing overt neurotoxicity or maternal toxicity. They examined both neurobehavioral and neurochemical outcomes, showing that exposure on gestational day (GD) 8 or 15 significantly reduced pup to PND 21; while weight gain was impaired only in pups exposed on GD 15; by PND 40, however, no differences in body weight were observed. Motor performance assessed by the Open-Field Test at PND 40 revealed a significant reduction in the number of rearings (both at GD 8 and GD 15), suggesting impaired exploratory behavior and possible alterations in cerebellar circuitry. Using a Novel Object Exploration Test, the authors found that MeHg-exposed rats spent less time exploring novel objects than controls, indicating deficits in nonspatial working memory. This reduction in exploratory activity could not be attributed to anxiety, since no alterations were observed in the Elevated Zero-Maze Test. Neurochemical analysis in cortical cell cultures from 1-day-old pups further revealed increased basal extracellular glutamate levels in MeHg-treated groups, indicating heightened neuronal sensitivity to excitotoxic injury. These findings are consistent with Baraldi et al. (57), who reported that a single oral dose of MeHg (8 mg/kg at GD 8) impaired exploratory activity, learning, and memory at PND 60, alongside alterations in the gene expression of a specific NMDA receptor subunit (NR2B) in the hippocampus.

Ferraro et al. (69) confirmed excitotoxic injury in primary neuronal cultures derived from the cerebral cortex of 1-day-old pups born to dams treated with MeHg on GD 15 (4 mg/kg). They demonstrated reduced cell viability and increased apoptotic cell death, suggesting greater neuronal vulnerability to excitotoxic insult. In cultures from the 8 mg/kg exposure group, the damage was more extensive, including necrotic death of cortical neurons and degeneration of neuritic processes. Behavioral testing further revealed a correlation between this severe early cortical neuron damage and long-term memory impairment, as observed in the Passive Avoidance Task at PND 90 in male rats prenatally exposed to 8 mg/kg of MeHg. This correlation was not detected in the 4 mg/kg group. In addition, the higher dose exposure was linked to general toxicity, including increased mortality at birth and reduced growth before weaning. Consistent with these findings, Maia et al. (71, 72 reported behavior alterations in motor activity, anxiety, panic, and depression in both male and female rats evaluated at PND 60 following prenatal exposure to MeHg (8 mg/kg, GD 15).

Sakamoto et al. (58) demonstrated that a diet containing 5 ppm of MeHg, administered to rat dams from 8 weeks before conception through gestation, and lactation, and continuously to young male offspring until PND 60, was associated with abnormal cerebellum development and behavioral deficits in motor coordination (Rotarod Test) and learning abilities (Passive Avoidance Test) evaluated at PND 35 and PND 42. In contrast, Goulet et al. (60), using 4, 6, or 8 ppm MeHg in the drinking water to dams from GD 2 until PND 21, did not observe impairments in motor coordination learning at PND 42 (Rotarod Test). However, they reported significant alterations in spontaneous locomotion and rearing behavior (Open-Field Test) at PND 63, as well as working memory deficits (modified T Maze) at PND 70. Similarly, Roegge et al. (62) examined offspring of dams exposed to 0.5 ppm MeHg in the drinking water from 4 weeks before mating until PND 16 and found only subtle effects, limited to female offspring, who showed impaired performance in the Rope Climb Test on the third day of testing with the thinnest rope at PND 60.

Newland et al. (61) evaluated offspring from dams exposed to 0.5 or 6.5 ppm MeHg in the drinking water from 4 weeks before mating until PND 16, assessing them in adulthood (1.7 years old) and aging (2.3 years). They found that developmental exposure to MeHg produced long-lasting behavioral effects, including delayed acquisition of choice behavior and reduced sensitivity to changes in reinforcement rates in operant conditioning tasks. Weiss et al. (63) also demonstrated behavioral consequences of perinatal and lifetime MeHg exposure in male mice. Motor deficits were predominantly observed at 5, 15, and 26 months in both perinatal and lifetime exposure groups, while delayed spatial response alternation was observed at 22 months (with the exception of the 1 ppm perinatal exposure group). In addition, running in a wheel to earn food pellets (schedule-controlled operant behavior) was reduced in the 3 ppm lifetime exposure group at 5 months. Using a similar model, Sugawara et al. (68) reported that dietary exposure to 5 ppm of MeHg in female mice, beginning 4 weeks before mating and continuing throughout gestation and lactation, induced early preweaning developmental changes (Grasp Reflex and Negative Geotaxis) in both sexes. Furthermore, male offspring exhibited motor and learning impairments at 8 weeks of age.

Although prenatal and lactational exposure models to MeHg are commonly associated with motor deficits, postnatal exposure may instead lead to hyperlocomotor activity. Stringari et al. (73) administered daily subcutaneous injections of MeHg (7 mg/kg body weight) to male and female Swiss albino mice during four early postnatal windows: PND 1-5, PND 6-10, PND 11-15, or PND 16-20. When assessed at PND 35, mice exposed during the second half of the suckling period (PND 11-20) showed a significant increase in spontaneous locomotor activity in the Open-Field Test. This hyperactivity was linked to cerebellar oxidative stress, as evidenced by increased lipid peroxidation (TBARS levels), decreased glutathione levels, and reduced glutathione-related enzymes (glutathione peroxidase-GPx and glutathione reductase-GR).

Oxidative stress has also been implicated as a mechanism underlying behavioral changes observed in prenatal and lactational exposure models. Using an *in vitro* system, Onishchenko et al. (66) exposed primary neural stem cells (NSCs) cultured derived from adult AREhPAP transgenic mice brains to MeHg (0.1 or 0.5 µM/24 h), demonstrating that oxidative stress could mediate MeHg-induced behavioral effects. *In vivo*, reduced monoamine oxidase (MAO) activity in the brainstem of adult female offspring was associated with altered auditory startle response. In this model, dams were treated with 0.5 or 1.0 mg MeHg/kg/day by gavage from 4 weeks before mating until GD 20, resulting in a dose-dependent increase in whole-brain Hg concentrations (ng/g) in offspring measured at PND 41: Ctrl male group = 5.3 ± 1.8; 0.5 mg/kg/day male group = 20.2 ± 1.6; 1.0 mg/kg/day male group = 58.2 ± 3.6; Ctrl female group = 9.2 ± 3.5; 0.5 mg/kg/day female group = 34.6 ± 3.2; 1.0 mg/kg/day female group = 45.5 ± 5.1) (Xiao et al., 2006).

Montgomery et al. (67) investigated behavioral outcomes in adult offspring developmentally exposed to chronic, low-dose MeHg at levels more comparable to human dietary ingestion. Pregnant C57Bl/6J mice were fed food containing MeHg from GD 8–18, corresponding to an estimated daily dose of 0.01 mg/kg body weight. Offspring were assessed for motor and coordination abilities at 2 months of age and for spatial learning in the Morris Water Maze at 6 months. Except for rearing behavior in the Open-Field Test, no sex-specific effects were detected. Female offspring at 2 months showed a reduced number of rearing compared to males. Although cerebellar mercury content did not differ between exposed and control mice at 2–3 months of age, MeHg-exposed offspring exhibited an overall decrease in coordination and impaired motor learning, suggesting that prenatal exposure to MeHg led to developmental neurobiological alterations in cerebellar circuitry. Additionally, MeHg-exposed mice spent less time in the center of the Open Field Test, consistent with increased anxiety-like behavior. At 6 months, spatial learning deficits were observed in the Morris Water Maze, independent of the impaired motor deficits observed at 2 months, as swimming performance in the maze was comparable between exposed and control mice.

Unfortunately, research on the effects of EDCs such as MeHg on maternal behavior remains limited. Carratù et al. (65) described no symptoms of neurotoxicity or maternal toxicity in dams exposed to MeHg (8 mg/kg) on GD 8 or 15. Reproductive parameters evaluated showed dam weight gain, dams giving birth, pregnancy length (days), and litter size at birth were similar between control and MeHg-treated groups. However, postnatal mortality of male pups was significantly increased in both MeHg-treated groups, and dams have not been evaluated in any behavioral test. In other studies (59, 61–63), the dams and pups’ toxicological parameters evaluated included only breeding and litter outcomes.

**Figure 2:**
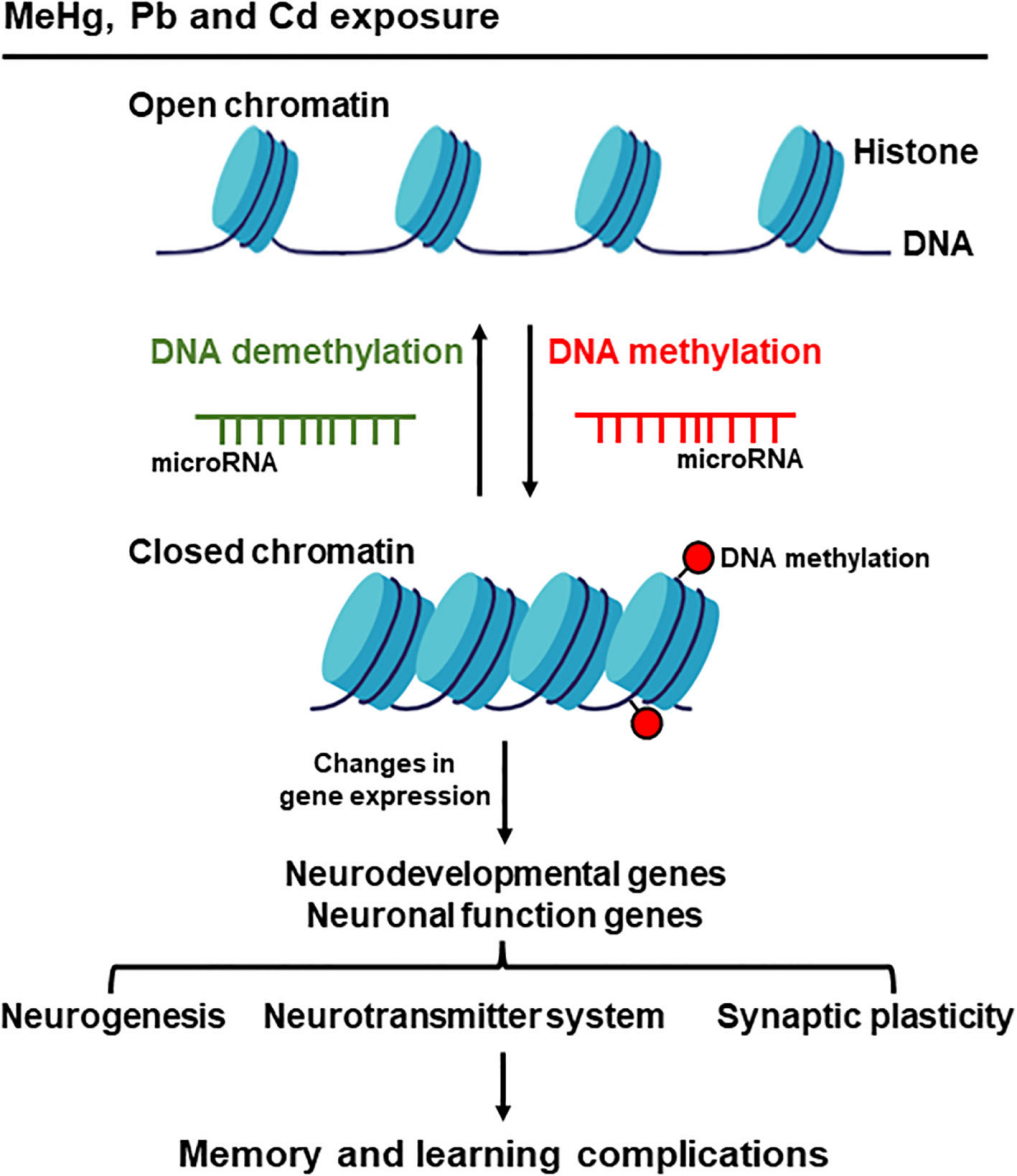
Epigenetics changes in the gene expression without alteration in DNA sequence. It mainly consists of DNA methylation, histone modification, non-coding RNA (microRNA), chromatin remodeling after exposure to MeHg, Pb and Cd. This exposure could modified neurogenesis, neurotransmitter system, synaptic plasticity and function related to memory and learning complications.

Investigations also reported MeHg exposure was linked to epigenetic changes in human and experimental models (74–77) (**Figure 2**). MeHg exposure *in utero* was associated with differential methylation of CpG sites detectable in peripheral blood of children and altered methylation patterns correlated with poorer performance on standardized cognitive tests, implicating disrupted epigenetic programming of neurodevelopmental genes as a mediator of memory and learning deficits later in life. These persistent DNA methylation alterations may affect neural transcriptional networks critical for synaptic plasticity and hippocampal function, consistent with observed cognitive effects in epidemiological cohorts with environmental MeHg exposure (74, 76). MeHg exposure perturbs major epigenetic regulatory mechanisms during neurodevelopment, including DNA methylation, histone modifications, and altered expression of chromatin regulators, targeting genes involved in neuronal differentiation and synaptic plasticity such as Brain-Derived Neurotrophic Factor (BDNF) (75). These MeHg-induced epigenetic disruptions coincide with persistent deficits in learning and memory in rodents exposed perinatally or during early development. Mechanistically, exposure alters DNA methyltransferase (Dnmt) and histone deacetylases (HDAC) activity, histone marks, and microRNA expression, which can durably reprogram neurodevelopmental gene expression, hinder neurogenesis, and impair synaptic plasticity pathways essential for memory formation (77).

Thus, developmental exposure to MeHg produces a wide spectrum of long-lasting neurobehavioral disturbances, with severity depending on dose, timing, and duration of exposure. Prenatal and early postnatal MeHg exposure consistently disrupts motor behavior, learning, and exploratory activity, with several studies highlighting sex-specific vulnerability—particularly in males, who frequently exhibit impaired dopaminergic function, reduced exploratory behavior, learning and memory deficits, and increased susceptibility to depressive-like phenotypes. Collectively, these findings demonstrate that MeHg acts as a potent developmental neurotoxicant.

### 2.3 Pb EDCs properties

Pb exposure disrupts multiple hormone systems, particularly the HP axis, resulting in blunted Thyroid-stimulating hormone (TSH), growth hormone (GH), and FSH/LH responses to stimulation with thyroid-releasing hormone (TRH), growth hormone-releasing hormone (GHRH), and gonadotropin-releasing hormone (GnRH), respectively (78). These alterations can contribute to reduced thyroid function, imbalances in sexual hormones, and reproductive dysfunction (79). Pb also interferes with hormone synthesis, transport, and binding, negatively impacting overall health, neurodevelopment, and growth. Consequently, Pb-induced endocrine disruption has been linked to neurodevelopmental and behavioral impairments, reproductive disorders, and immune system disturbances (80–82). In addition, a positive correlation between the blood lead levels (BLLs) and parathyroid hormone (PTH) has been reported in adolescents, likely reflecting Pb’s disruptive effects on calcium and vitamin D metabolism and its contribution to increased bone turnover (83).

The hypothalamic-pituitary-adrenal (HPA) axis is the body’s primary stress response system. Pb exposure is associated with dysregulation of the HPA axis, including altered glucocorticoid (cortisol) levels and increased allostatic load. Early-life exposure was linked to changes in both diurnal and reactive cortisol levels in children, whereas adult exposure was associated with both increased cortisol reactivity and greater allostatic load. Although the evidence supports a role for Pb in disrupting HPA axis function, further research is needed to fully understand the extent to which this dysfunction mediates Pb’s adverse health effects across the life course (84).

Pb exposure has also been linked to pineal gland dysfunction. The pineal gland’s primary function is to produce and release melatonin, which controls the sleep-wake cycle (85). Pb exposure can disrupt this process, potentially reduce melatonin levels, and disturb circadian rhythms. Since melatonin exerts antioxidant, free radical-scavenging, anti-apoptotic, anti-inflammatory, and neuroprotective effects, it’s potential to counteract Pb-induced cognitive deficits, anxiety, and depressive-like behavior has been investigated (86). In co-exposure studies, melatonin treatment not only decreased Pb levels in the blood and organs but also enhanced its excretion in urine and feces (87). Building on this, Soto-Arredondo et al. (88) examined the effects of Pb and melatonin co-administration on the expression of metal transporter proteins (ZIP8 and ZIP-14- zinc transporter solute carrier family 39 member 8 and 14, respectively, CTR1- high-affinity copper-uptake protein 1, and DMT1- dimethyladenosine transferase 1) in the liver and kidney. They found that melatonin reduced DMT1 protein expression in the kidney, which corresponded with lower Pb levels in the blood and kidney of exposed rats.

Inorganic and organic Pb compounds are classified as “probably carcinogenic to humans” (Group 2A) (89). Animal data support Pb’s carcinogenic potential, showing tumor development at multiple tissue sites and via different exposure routes. Epidemiological studies have suggested associations between Pb exposure and elevated risks of stomach, lung, kidney, brain, and meningeal cancers. However, this human evidence remains inconsistent, with many associations considered weak (90). Mechanistically, Pb can act as a metalloestrogen, mimicking endogenous estrogen by binding to estrogen receptors, which may contribute to hormone-dependent cancers and other abnormalities related (91–93).

Pb exposure, particularly during the prenatal period, has been linked to an increased risk of obesity in children. Evidence in adults, however, is inconclusive, with studies reporting positive, negative, or no significant associations (94–96). Proposed mechanisms include Pb-induced alterations of the gut microbiome, epigenetic gene regulation, and promotion of oxidative stress (97–99). Interestingly, adequate maternal folate levels may offer protection against Pb-induced obesity in offspring and other related abnormalities (100).

Environmental Pb exposure is linked to a higher risk of type 2 diabetes by disrupting glucose metabolism and pancreatic beta-cell function by oxidative stress mechanisms (101–103). The results of a five-year, repeated-measures study of Chinese adults revealed that an increase in BLLs was associated with impaired glucose homeostasis, particularly in women. This led to elevated fasting plasma glucose and beta-cell dysfunction, as indicated by a decreased homeostasis model assessment of beta-cell function (HOMA-B) (104). However, studies showed associations between elevated BLLs and increased risk or poorer control of type 2 diabetes, some studies in general populations have yielded conflicting results (102, 105). Besides, the risk can be compounded by co-exposure to Cd, and Pb exposure may accelerate diabetic complications similar observed in nephropathy (103).

Arsenic (As), Cd, Pb, and Hg exposure were positively associated with metabolic syndrome consequences. When analyzing individually, significant positive associations were observed for As, Pb, and Hg, whereas the association for Cd was positive but less consistent and influenced by factors such as sex (106). More recently, Hasani et al. (107) reported significant associations between blood levels of Cd, Hg, and Pb and increased odds of metabolic syndrome. Thus, Pb acts as a pervasive EDC that impairs multiple hormonal axes, contributing to thyroid dysfunction, reproductive hormone imbalance, and disrupted growth and development. Pb further interferes with hormone synthesis, transport, and binding, linking exposure to neurodevelopmental, reproductive, immune, and calcium-vitamin D disturbances, cancer, stress, gut microbiome, and glucose metabolism and diabetes (**Figure 1**).

### 2.4 Pb perinatal exposure and behavior effects

Perinatal Pb exposure has been associated with abnormal vocalizations, delayed sensorimotor reflexes, early locomotor abnormalities, neuronal damage, deficits in learning, memory, and cognitive flexibility, increased anxiety- and depressive-like behaviors in various exposure models (**Figure 1**, **Table 2**). Preweaning neurobehavioral parameters were evaluated in the Pb-exposed offspring (112, 126, 127, 129). De Marco et al. (112) evaluated the ultrasonic vocalization (USV), an early behavior essential for rat pup development, in offspring of unspecified sex at PND 7 and 14. BLLs at PND 7 ranged from 5.7 to 36.5 µg/dL. They observed that Pb exposure during pregnancy and/or lactation led to a dose-dependent decrease in USV in 7-day-old pups, whereas exposure during pregnancy resulted in a dose-dependent increase in USV in 14-day-old pups. Additionally, locomotor activity was augmented in all Pb-exposed offspring at PND 14.

**Table 2:** Pb perinatal exposure schemes.

Species	Dose of Pb used and route of administration	Exposure period	Time of behavioral analyses in the offspring*	Reference
Wistar rats	500 ppm of lead acetate, oral route by drinking water	GD 0 - PND 21	PND 23 PND 70	Moreira et al. (108)
Wistar rats	0.03% (Low), 0.09% (Middle), or 0.27% (High) lead acetate, oral route by contaminated food	GD 0 - GD 21	PND 49	Yang et al. (109)
Wistar rats	10 mg/kg Pb acetate and 13.5 mg/kg Na acetate, oral route by gavage	GD 0 - PND 22 Groups: Na–Na: pups were kept with their Na-treated dams during lactation Pb–Pb: pups were kept with their Pb-treated dams during lactation Na–Pb: pups were cross-fostered to a Pb-treated dam at birth Pb–Na: pups were cross-fostered to a Na-treated dam at birth	PND 70	de Souza Lisboa et al. (110)
Wistar rats	0.2% Pb acetate, oral route by drinking water	PND 1 - PND 30	PND 60 (locomotor activity and anxiety testing) PND 80 (contextual fear conditioning)	Jaako-Movits et al. (111)
Wistar rats	8, 16 and 24 mg/kg Pb acetate, oral route by gavage	30 days prior to breeding until PND 21 (only female rats) Groups: Pregnancy Pregnancy + Lactation Lactation	PND 7 PND 14 (No sex specification)	de Marco et al. (112)
C57BL/6 mice	27 (0.005%, low), 55 (0.01%, moderate), or 109 ppm (0.02%, high) of lead acetate, oral route by drinking water (GLE model) 27 (0.005%, low) or 55 ppm (0.01%, moderate) of lead acetate, oral route by drinking water (PLE model)	Gestational Lead Exposure (GLE) model: 2 weeks prior to mating until PND 10 (only female rats) Postnatal Lead Exposure (PLE) model: PND 0 - 21	12 months of age	Leasure et al. (113)
BALB/cAnNTac mice	20 ppm (0.1 mM Pb acetate), oral route by drinking water	GD 8 - PND 21	Adult life (without specifying the days of behavioral analysis related to exploratory/anxiety measures and Morris Water Maze) 10 months of age in the analysis of aggressive behavior	Kasten-Jolly et al. (114)
Long Evans rats	250 ppm, 750 ppm or 1500 ppm Pb acetate, oral route by contaminated food	10 days prior to breeding until PND 21 (only female rats) Observation: At weaning, 1 male and 1 female from each litter were combined with animals from other litters to form behavioral cohorts. Rats were either housed 3 to a cage with no added stimuli (non-enriched) or 6 to a cage containing a variety of toys, climbing and nesting materials, and tunnels that were changed twice per week (enriched group)	PND 55	Anderson et al. (115)
Sprague- Dawley rats	10 µg/mL Pb acetate trihydrate, oral route by drinking water	GD 0 - PND 20	PNDs 1 - 10 (no sex specification) PNDs 21 - 25 PNDs 56 - 60	Betharia & Maher (116)
Wistar rats	2 mM Pb chloride, oral route by drinking water	3 weeks before mating (only female rats)-PND 21: Pre-weaning Pb group PND 21 - PND 84: Post-weaning Pb group	PNDs 85 - 90 (male and female analyzed together)	Xiao et al. (117)
C57BL/6J mice	300 ppm Pb acetate, oral route by drinking water	GD 1 - GD 10.5	12 weeks of age until 20 weeks of age	Hill et al. (118)
Wistar rats	0.2% Pb acetate added 0.5 ml/l glacial acetic acid, oral route by drinking water	Gestation lead exposed (G): GD 1 - 21 Lactation lead exposed (L): PND 1 - 21 Gestation and lactation lead exposed (GL): GD 1 - PND 21 Pre-gestation lead exposed (PG): 30 days prior to breeding (only female rats)	PNDs 26 - 36	Barkur & Bairy (119)
Wistar rats	0.2% Pb acetate added 0.5 ml/l glacial acetic acid, oral route by drinking water	Gestation lead exposed (G): GD 1 - 21 Lactation lead exposed (L): PND 1 - 21 Gestation and lactation lead exposed (GL): GD 1 - PND 21 Pre-gestation lead exposed (PG): 30 days prior to breeding (only female rats)	Preweaning neurobehavioral parameters	Barkur & Bairy (120)
Long Evans rats	150 ppm, 375 ppm or 750 ppm Pb acetate, oral route by contaminated food	Perinatal (PERI) exposure: 10 days prior to breeding until PND 21 (only female rats) Early postnatal (EPN) exposure: PND 1 - 21 Long-term postnatal (LPN) exposure: PND 1 - 55	PND 55	Anderson et al. (121)
Wistar rats	0.2% Pb acetate, oral route by drinking water	PND 1 - 21	PND 30 PND 45	Rahman et al. (122)
Wistar rats	0.2% Pb acetate, oral route by drinking water	GD 7- 28 weeks of age in the permanent (PbP) group GD 7- 28 weeks of age in the intermittent (PbI) group (period of lead abstinence for 8 weeks and again a period of lead exposure of 8 weeks)	27 weeks of age (No sex specification)	Shvachiy et al. (123)
Wistar rats	0,15% Pb acetate, oral route by drinking water	GD 6 until weaning	PND 61	Chintapanti et al. (124)
FVB mice	500 ppm Pb acetate, oral route by drinking water	PND 0 - 23	PND 23 PND 60	Ortega et al. (125)
Sprague-Dawley rats	0.3% Pb acetate, oral route by drinking water	2 weeks prior mating (only female rats) until PND 21	PND 19	Nam et al. (126)
Wistar rats	5 mg/kg/day, oral route by drinking water	4 weeks prior mating (only female rats) until PND 23	PNDs 4, 7, 10, 12, and 13 (neonatal stage); PNDs 30, 35, 60, 63-65, and 68-72 (juvenile/adult stage)	Tartaglione et al. (127)
Wistar rats	100 ppm Pb acetate, oral route by drinking water	PND 1 - 21	PND 90 (No sex specification)	Shirke et al. (128)

Pb, lead; GD, gestational days; PND, postnatal days; Na, sodium. *Male and female separated or as described in the line.

In male offspring exposed to 0.2% Pb in drinking water under four different protocols: gestation period (G group), lactation period (L group), both gestation and lactation periods (GL group), and for 1 month before pregnancy (PG group)- early neurobehavioral and sensorimotor reflex development were impaired between PND 6 and 8, as measured by Surface Righting Reflex, Swimming Development, Negative Geotaxis, and Ascending Wire Mesh Test. BLLs at PND 22 ranged from 3.03 to 31.59 µg/dL (129). Learning and spatial memory deficits were subsequently observed at PND 26–36 in the Passive Avoidance and Morris Water Maze test (119). These behavioral alterations have been linked to oxidative stress in the hippocampus, cerebellum, and frontal cortex of rat pups at PND 30, indicated by increased TBARS levels and glutathione peroxidase activity, along with decreased levels of reduced glutathione (130). Histological analysis at PND 30 revealed extensive neuronal damage in the hippocampus, amygdala, and cerebellum of the L group, comparable to that in the GL group. The G group exhibited less degeneration, while the PG group showed no detectable neural damage (120).

Nam et al. (126) analyzed locomotor coordination in male pups at PND 19, using the Bar Holding Test and Wire Mesh Ascending Test, with female pups included to maintain equal litter sizes. BLLs at PND 22 were ≈ 200 µg/dl. Male pups exhibited locomotor deficits in both tests. Interestingly, gintonin, a novel G protein-coupled lysophosphatidic acid receptor (LPAR) ligand isolated from ginseng, was found to protect the developing rat cerebellum from Pb-induced impairments. This neuroprotection involved modulation of multiple marker proteins, including Purkinje cell integrity, apoptosis (Bax and Bcl-2), Gamma-aminobutyric acid (GABA) synthesis and transport (GAD and GABAT1), LPA1 and N-methyl-D-aspartate (NMDA) receptor (post-NMDAR1), oxidative stress (Nrf2, Mn-SOD, IL-1β, and TNFα), synaptic function (synaptophysin), myelination (MBP, Olig-2), and neurotrophic signaling (Sirt-1, BDNF).

Tartaglione et al. (127) demonstrated alterations in motor, emotional, and cognitive functions in offspring of both sexes exposed to Pb, with median BLLs of 0.255 µg/mL. Behavioral assessments were conducted at PNDs 4, 7, 10, 12, and 13 (neonatal stage) and at PNDs 30, 35, 60, 63-65, and 68-72 (juvenile/adult stage). At PND 23, Pb concentrations in the brain reached median values of 0.452 µg/g in the cerebral cortex and 0.685 µg/g in the hippocampus. During the neonatal stage, both male and female pups displayed increased “stereotyped” explorative behaviors (head rising, wall climbing) at the expense of locomotion, suggesting impaired inhibitory control and perseveration. These changes were accompanied by reduced latency in the righting reflex and Negative Geotaxis. At the juvenile stage, the mild motor impairments observed in the first ten days of life had been resolved, likely due to enhanced sensory and motor integration with age. At this age, both sexes showed reduced arm entries in the Y-Maze Test, suggesting a deficit in explorative behavior. In adulthood, however, Pb effects became sex-specific: females exhibited increased anxiety in the Elevated-Plus Maze and cognitive deficits in the Morris Water Maze. Mechanistic studies pointed to disruptions in glutamatergic signaling, particularly in the expression and trafficking of NMDA and α-amino-3-hydroxy-5-methyl-4-isoxazolepropionic acid (AMPA) receptor subtypes, which are critical for learning and memory. Specifically, Pb exposure increased post-synaptic expression of GluN2A and GluA1 subunits of glutamate receptor in female hippocampal spines at PND 23. The authors suggested that these molecular alterations, together with possible Pb-induced interference in hormonal or neuroendocrine pathways, may underline the observed sex-specific behavioral differences at environmentally relevant doses.

Additionally, Betharia & Maher (116) demonstrated that female offspring were more sensitive to Pb-induced deficits in learning and memory, as assessed in the Morris Water Maze Test, and also showed higher body weight gain. Their exposure model produced lower BLLs and Pb brain concentrations compared to Tartaglione et al. (127). Offspring from dams exposed to 10 µg/mL Pb acetate trihydrate in drinking water exhibited Pb levels of 5.04 (± 0.70) ng/g in whole brain and 6.22 (± 1.92) ng/g in hippocampus at PND 25, and 1.99 (± 1.01) ng/g at PND 60. Corresponding BLLs were 85.17 (± 8.33), 9.21 (± 1.13), and 0.30 (± 0.11) ng/g at PNDs 2, 25, and 60, respectively. Depressive-like behavior in adulthood (PND 70) was previously demonstrated in female offspring following gestational and lactational exposure to Pb acetate (10 mg/kg), which resulted in BLLs of 6.8 (± 1.5) µg/dL (110). Similarly, Tartaglione et al. (127) also showed an increase in susceptibility to anxiety and depressive-like behavior as well as learning and memory impairments in females. Interestingly, in a transgenerational model, F3 female offspring showed reduced fear or anxiety-like behavior but continued to display impaired learning and memory (131).

Moreira et al. (108) examined behavioral changes in male offspring from dams exposed to 500 ppm of Pb acetate in drinking water from GD 0 to PND 21, identifying deficits in motor, social, and learning abilities. At PND 23, male offspring showed a significant increase in ambulation in the Open-Field Test and impaired learning and memory in the Shuttle Box, effects that persisted at PND 70, along with a trend toward anxiety-like behavior and impaired social abilities in the Social Interaction Test. Similarly, spatial learning deficits were detected in both female and male offspring at PND 49 following gestational exposure (GD 0-GD 21) to Pb acetate in the contaminated food at low (0.03%), medium (0.09%), or high (0.27%) concentrations (109). Social impairments in male offspring were also observed when exposure occurred only during early gestation (GD 1-GD 10.5), with 300 ppm Pb acetate in drinking water, a model that revealed altered cytosines followed by guanine residues (CpG) island methylation in promoter regions and associated changes in gene expression in the frontal cortex (118). Kasten-Jolly et al. (114) demonstrated that both male and female mice exposed to even the lowest Pb levels displayed significant impairments in learning and memory in adulthood. Additionally, male offspring at 10 months of age showed increased aggressive behavior, which was linked to changes in vomeronasal receptor gene (V2r12) expression, a gene associated with odor detection and social behavior.

Male mice offspring exposed under two different models, a gestational lead exposure (GLE) model (2 weeks before mating until PND 10) and a postnatal lead exposure (PLE) model (PND 0-21), to Pb acetate in drinking water at 27 ppm (0.005%, low), 55 ppm (0.01%, moderate), or 109 ppm (0.02%, high) displayed exploratory and motor deficits when evaluated at 12 months of age, whereas female offspring showed no behavioral changes. In addition, male offspring from the GLE group exhibited late-onset obesity and neurochemical alterations in the striatum and forebrain, specifically changes in dopamine (DA) and its metabolite 3,4-dihydroxyphenylacetic acid (DOPAC) concentrations (113). Complementing these findings, Chintapanti et al. (124) reported a range of motor, exploratory, and cognitive impairments, along with altered responses to analgesia (Hot Plate Test), in male rat offspring from dams exposed to 0.15% Pb acetate in drinking water from GD 6 until weaning. These behavioral alterations were associated with oxidative stress and changes in the acetylcholinesterase activity across the hippocampus, cerebellum, cerebral cortex, and medulla.

Shvachiy et al. (123) showed that offspring exposed to 0.2% Pb acetate in drinking water continuously from GD until 28 weeks of age (permanent exposure, PbP group) or intermittently (PbI group, 8 weeks of exposure, 8 weeks of abstinence, followed by another 8 weeks of exposure) exhibited behavioral changes in adulthood. At PND 189, both groups displayed anxiety-like behavior in the Elevated-Plus Maze Test, while the PbP group also showed hyperactivity in the Open-Field Test. BLLs were 24.4 ± 4.9 μg/dL in the PbP group and 18.8 ± 2.0 μg/dL in the PbI group. Proposed mechanisms underlying Pb-induced toxicity in these models’ included hypertension, increased respiratory frequency and chemoreflex sensitivity, baroreflex impairment, decreased synaptic activity, neuroinflammation, and reactive gliosis in the hippocampus. Hippocampal damage (CA1, CA3, and dentate gyrus) was also implicated in pre-, during and post-weaning Pb exposure models, which promoted anxiety-like behavior, learning and spatial memory impairments detected in adulthood (117, 128). Consistently, Jaako-Movits et al. (111) demonstrated that male offspring from dams exposed to 0.2% Pb acetate in drinking water (PND 1-30) showed long-lasting or even permanent alterations in brain function, including anxiety-like behavior at PND 60 and impaired contextual fear conditioning at PND 80, despite significantly reduced BLLs at these time points (≈ controls). BLLs (µg/dL) were 29.3 ± 5.0 (PND 15), 34.2 ± 5.8 (PND 30), 10.4 ± 3.2 (PND 60), and 6.5 ± 1.2 (PND 80), while the brain levels (ng/g) were 456 ± 23 (PND 15), 781 ± 87 (PND 30), 20 ± 8 (PND 60), and 6 ± 1 (PND 80).

Anderson et al. (115) showed that behavioral outcomes related to learning and memory evaluated by the Morris Water Maze Test, were influenced by sex, rearing environment, and Pb exposure, with complex interactions among these factors. In this model, dams were fed Pb-containing food (0, 250, 750, or 1500 ppm of lead acetate) for 10 days before breeding and remained on the same diet through weaning. Litters were then separated in the behavioral courts and maintained in either enriched or non-enriched environments until evaluation at PND 55. During the acquisition phase, all groups improved path efficiency across training, but enrichment had pronounced effects in the first two days: enriched male rats located the platform more efficiently regardless of Pb exposure. Across the remaining training days, enrichment provided a significant advantage only at 750 and 1500 ppm Pb exposure. In females, enrichment enhanced path efficiency compared to non-enrichment during the first six training days in controls, but this advantage was progressively diminished with Pb exposure, partially at 250 and 750 ppm, and absent at 1500 ppm Pb. In the probe trial, enriched male rats showed better performance than non-enriched males only at 750 ppm Pb, while swim speed was significantly reduced only in enriched males exposed to 1500 ppm. In females, significant enrichment-related advantages in path efficiency were seen at 250 and 750 ppm Pb, with swim speed reductions noted in non-enriched females only at 250 ppm. Overall, the authors concluded that in females, developmental Pb exposure combined with a non-enriched environment impaired spatial learning, resulting in slower and less efficient performance. In males, enrichment conferred an early advantage in task acquisition, but this effect was largely absent at later stages regardless of Pb exposure.

Using a different paradigm, Trace Fear Conditioning, a task requiring coordinated activity of both the medial prefrontal cortex and hippocampus, Anderson et al. (121) assessed the effects of Pb exposure on associative learning and memory. Dams received contaminated food containing 150, 375, or 750 ppm Pb acetate during three exposure windows: perinatal (PERI; 10 days before breeding until PND 21), early postnatal (EPN; PND 1-21), or long-term postnatal (LPN; PND 1-55). The study found no intrinsic sex differences in the ability to learn and consolidate associative memory across exposure windows or Pb doses. However, Pb exposure impaired long-term memory retention and recall in both sexes.

Rahman et al. (2018) suggested that quinolinic acid (QA), a downstream metabolite of tryptophan through the kynurenine pathway and known excitotoxin acting via N-methyl-D-aspartic acid receptor (NMDAR) activation, in Pb-induced neurotoxicity. In their model, pups were exposed through dams’ drinking water (0.2% Pb acetate) from PND 1 to 21 and assessed in the Morris Water Maze Test at PND 30 and PND 45. Early postnatal Pb exposure significantly increased circulating QA levels, elevated the number of QA-immunoreactive cells in the brain, and impaired learning and short-term memory. To confirm causality, a second experiment used direct intraventricular infusion of QA into 21-day-old rats for 7 days. This treatment reproduced the learning and short-term memory deficits observed with Pb exposure and was associated with reduced levels of synaptic signaling proteins involved in learning and memory (PSD-95, PP1, and PP2A), and increased Tau phosphorylation, potentially contributing to microtubule disassembly.

In a model investigating Pb exposure during the lactation period, dams received 500 ppm of Pb acetate in their drinking water from PND 0 to 23. Pb accumulated in the hippocampus, striatum, cortex, and cerebellum of offspring, reaching 23 ± 0.8, 26.7 ± 5.6, 24.1 ± 4.4, and 23.4 ± 2.8 μg Pb/mg of tissue, respectively. At PND 23, offspring displayed reduced locomotor activity, and by PND 60, they exhibited impaired memory consolidation, without changes in acquisition (Buried Food Location Test). These behavior alterations were associated with oxidative stress in multiple brain structures (hippocampus, striatum, cortex, and cerebellum) and with dysregulation of the kynurenine pathway (125).

Dou et al. (132) investigated cortical neuron-specific cell populations in adult mice (10 months of age) exposed *in utero* to Pb via maternal drinking water at 2.1 ppm (low) or 32 ppm (high) Pb acetate, corresponding to peak maternal blood levels of approximately 2.5 and 25 µg/dL, respectively. Exposure was terminated at weaning. They observed a weak association between early-life Pb exposure and DNA hypomethylation, with several affected genes linked to neurodevelopment and cognitive function. In the high exposure group, differentially methylated regions (DMRs) were enriched for pathways related to response to X-ray, positive regulation of hormone secretion, and neural tube formation, while depleted for pathways such as positive regulation of hair follicle development and positive regulation of cholesterol biosynthesis. In the low exposure group, DMRs were enriched for pathways including female pregnancy, neuropeptide signaling, lipid metabolism, and carbohydrate biosynthesis.

Investigations also reported Pb exposure was linked to epigenetic changes in human and experimental models (133–136) (**Figure 2**). In human studies, prenatal lead exposure is associated with gene-specific DNA methylation changes in umbilical cord blood that statistically mediate adverse cognitive and behavioral outcomes in early childhood. For example, trimester-specific maternal blood Pb levels were linked to differential methylation at genes such as coiled-coil serine rich protein 1 (*CCSER1)*, glucosaminyl (N-acetyl) transferase 1 (*GCNT1)*, trafficking protein particle complex subunit 6A *(TRAPPC6A)*, and vacuolar protein sorting-associated protein 11 (*VPS11)*, and these methylation marks partially mediated associations between lead exposure and decreased scores on infant neurodevelopment indices at 12–24 months. These findings support a model in which Pb alters the epigenetic landscape during neurodevelopment, contributing to deficits in learning, memory, and emotional regulation in offspring (133, 134). Pb exposure alters epigenetic regulators including DNA methylation, histone modifications, and non-coding RNAs, disrupting neuronal differentiation and plasticity pathways that underlie learning and memory. For instance, in rodent models of developmental lead exposure, aberrant DNA methylation and histone modification patterns have been reported in neural tissues, affecting expression of neurodevelopmental genes and synaptic function regulators. Altered epigenetic marks on genes involved in neurotransmitter systems and synaptic plasticity likely contribute to Pb-induced cognitive impairments observed in experimental learning and memory tasks (135, 136).

Thus, early-life Pb exposure consistently produces enduring neurobehavioral impairments, with effects shaped by exposure window, dose, sex, and environmental context. Preweaning assessments reveal disrupted ultrasonic vocalizations, delayed sensorimotor reflexes, and early locomotor abnormalities, accompanied by oxidative stress and neuronal damage across the hippocampus, cerebellum, amygdala, and frontal cortex. Juvenile and adult offspring show persistent deficits in learning, memory, and cognitive flexibility, increased anxiety- and depressive-like behaviors, and, in some cases, altered social interactions, aggression, and late-onset obesity. Sex-specific vulnerabilities frequently emerge, with female offspring showing heightened sensitivity to anxiety- and depression-related phenotypes and hippocampal glutamatergic disruption, whereas males more often display motor impairments, heightened aggression, and dopamine-related neurochemical changes. Mechanistic studies implicate oxidative stress, excitotoxicity via kynurenine pathway activation, disrupted synaptic signaling, altered NMDA/AMPA receptor expression, impaired neurotrophic and myelination pathways, and Pb-induced epigenetic reprogramming affecting genes involved in neurodevelopment, metabolism, and hormone regulation. Collectively, the evidence demonstrates that developmental Pb exposure—at both high and environmentally relevant levels—induces long-lasting and multi-domain neurobehavioral dysfunction through convergent molecular, neurochemical, and epigenetic pathways (137).

### 2.5 Cd EDCs properties

Cd is one of the most toxic and studied metalloestrogens, and it has been implicated in the development of estrogen-dependent diseases like breast cancer (91, 93). While experimental studies support estrogenic effects of Cd both *in vitro* and *in vivo*, evidence in human studies remains inconsistent (138). Cd is classified as a Group 1 human carcinogen by IARC, with established links to prostate and lung cancer and possibly kidney cancer (45) (**Figure 1**).

The signaling pathways mediating the transformation of benign prostatic hyperplasia (BPH) into malignancy have been investigated in the context of Cd exposure. Chronic Cd exposure (10 μM for 12 months) was shown to transform a normal immortalized human BPH cell line (BPH1) into malignant cells (CTBPH1). This transformation involved early activation (as soon as 2 months post-exposure) of metal regulatory element-binding transcription factor-1 (MTF1), which in turn upregulated zinc finger of the cerebellum 2 (ZIC2), an oncogenic transcription factor, and changed the cellular phenotyping (139, 140).

Cd exposure is also associated with an increased risk of diabetes and prediabetes. Elevated Cd levels in blood or urine are associated with metabolic syndrome, type 2 diabetes, and gestational diabetes (141–143,107, 144, 145). Mechanistically, Cd exposure impairs pancreatic beta-cell exacerbates glucose and lipid metabolism control. This disruption involves changes in lipid profiles, increased inflammation, and beta-cell dysfunction, ultimately contributing to insulin resistance and hyperglycemia (146).

Cd is a potential human obesogen and metabolic disruptor. Proposed mechanisms include disruption of adipose tissue physiology, resulting in impaired adipogenesis, altered lipid and carbohydrate metabolism in adipocytes, and adipose tissue endocrine dysfunction (141). Prenatal Cd exposure may also increase obesity risk. In the Newborn Epigenetics Study (NEST), the presence of Cd in maternal blood during pregnancy was associated with a higher risk of obesity in offspring at 5 years of age, independent of confounders such as Pb exposure and smoking status (147).

Cd and Pb frequently co-occur in the environment and are both linked to neurodevelopmental disorders and reduced birth weight. Low birth weight, followed by rapid postnatal weight gain, is a well-established risk factor for later cardiometabolic disorders, such as cardiovascular disease, type 2 diabetes, hypertension, and dyslipidemia (98). In a mouse model, exposure to 500 ppb CdCl_2_ in drinking water during a human gestationally equivalent period (GD0-PND10) resulted in obesity, fatty liver accumulation, and impaired glucose metabolism in adult female offspring (148).

Cd exposure is also associated with impaired reproductive health in both males and females. Reported effects include reduced fertility, disruption of sexual hormone synthesis, and damage to the germ cells and reproductive organs (143, 149, 150). In males, intergenerational adverse effects of Cd on Sertoli cells and reproductive development are primarily mediated through the follicle-stimulating hormone receptor (FSHR) pathway (149). In females, Cd exposure alters steroidogenesis across generations: in ovarian granulosa cells of the F1 generation, Cd downregulates steroidogenic acute regulatory protein (StAR), a critical cholesterol transport protein essential for hormone synthesis. In contrast, F2 offspring show upregulation of StAR along with increased expression of cytochrome P450 family 17 subfamily A member 1 (CYP17A1), cytochrome P450 family 19 subfamily A member 1 (CYP19A1), and steroidogenic factor 1 (SF-1), genes critical for steroid hormone synthesis and regulation (150).

Higher blood Cd levels are linked to greater Cd accumulation in the thyroid gland. This occurs because Cd has a strong affinity for cysteine-rich proteins such as metallothionein and glutathione within the thyroid, and has a very long biological half-life, allowing it to deposit and persist in the tissue (151). Elevated thyroid Cd levels are particularly evident in populations exposed to Cd-polluted environments or occupational settings (152). More recently, Shao et al. (26) demonstrated in two independent cross-sectional studies that higher Cd levels were consistently associated with sex-specific thyroid dysfunction.

Thyroid hormone (TH) disruption may contribute to Cd-induced neurotoxicity. In male Wistar rats, intraperitoneal Cd exposure either a single dose of 1 mg/kg or repeated treatments for 28 days at 0.1 mg/kg, with/without triiodothyronine (T3, 40 µg/kg/day) increased TSH levels, decreased T3 and T4, altered cholinergic transmission, and exacerbated neurodegeneration in basal forebrain cholinergic neurons, effects partially mediated by TH reduction. Cd-induced TH disruption also led to muscarinic 1 receptor (M1R) antagonism, overexpression of acetylcholinesterase S variant (AChE-S), downregulation of AChE-R, M2R, M3R, and M4R, and reduction of AChE and choline acetyltransferase activities (153). In the same exposure model, basal forebrain neurodegeneration was further associated with elevated levels of hydrogen peroxide (H_2_O_2_), malondialdehyde, TNF-α, IL-1β, IL-6, BACE1, Aβ, and phosphorylated-Tau, as well as decreased phosphorylated-AKT and phosphorylated-GSK-3β. These effects were partially reversed by T3 supplementation (154).

Thus, Cd is a highly toxic heavy metal that disrupts multiple endocrine pathways, contributing to estrogen-dependent diseases, metabolic dysfunction, reproductive impairment, TH disruption, and neuroendocrine toxicity. Cd can mimic or interfere with estrogen signaling, is classified as an IARC Group 1 carcinogen, and promotes malignant cellular transformation. Cd exposure is linked to metabolic syndrome, diabetes, obesity, and impaired β-cell and adipose tissue function. In the reproductive system, Cd reduces fertility and alters steroidogenesis across generations. Cd-induced alterations in thyroid hormones exacerbate neurotoxicity through oxidative stress, neuroinflammation, and impaired cholinergic signaling. Collectively, Cd acts as a potent EDC affecting metabolic, reproductive, thyroid, and neuroendocrine health.

### 2.6 Cd perinatal exposure and behavior effects

Cd perinatal studies, like MeHg and Pb, also involved various exposure models (**Table 3**). Offspring exposed to 10 ppm Cd in the drinking water of dams from GD 1 to PND 10 (156) or from GD 1 to GD 10.5 (118) showed no behavior alterations in motor function, nest building, marble burying, or social abilities. However, in brain tissue at PND 10, the expression of the thyroid hormone-related gene RC3 was inversely correlated with the ambulation distance in the Open-Field Test among female offspring. Additionally, Cd exposure disrupted sex hormone receptor mRNA expression: male offspring exhibited reduced estrogen receptor (ER) beta (ER-b) mRNA levels, while female offspring showed decreased ER-alpha (ER-a) and Progesterone receptor (PgR) mRNA levels 156) (**Figure 1**).

**Table 3:** Cd perinatal exposure schemes.

Species	Dose of Cd used and route of administration	Exposure period	Time of behavioral analyses in the offspring*	Reference
Wistar rats	0.3 or 0.6 mg Cd (CdCl2)/kg/day, injected subcutaneously	GD 7 - GD 15	Sensorimotor Development (PND 3-PND 7) PND 45/PND 90	Minetti & Reale (155)
C57BL/6J Jcl mice	10 ppm Cd, oral route by drinking water	GD 1 - PND 10	8 weeks of age	Ishitobi et al. (156)
Wistar rats	10 and 20 mg Cd/ Kg as cadmium chloride, oral route by gavage	GD 18 - 21 until 7th lactation day	Offspring behavior PND 21 PND 75	Couto-Moraes et al. (157)
C57BL/6J mice	10 ppm CdCl2, oral route by drinking water	GD 1 - GD 10.5	12 weeks of age until 20 weeks of age	Hill et al. (118)
C57BL/6 mice	10 mg Cd as cadmium chloride/L, oral route by drinking water	GD 0 - PND 21 (Cd pregnancy and lactation group) GD 0 - birth: adopted by a control (Cd pregnancy group) Birth from a control dam adopted by an exposed mother (Cd lactation group)	Prepuberty (3 weeks old) Puberty (5 weeks old) Young adulthood (7 weeks old) Adulthood (12 weeks old)	Zhao et al. (157)
Swiss albino mice	1.2 mg Cd/kg/day intraperitoneally (i.p.) Quercetin doses of 25, 50 and 100 mg/kg/ day, i.p.	GD 14 - GD 19	100-days (F1 generation) (No sex specification)	Halder et al. (158)
Swiss albino mice	1.2 mg Cd/kg/day intraperitoneally (i.p.) Quercetin doses of 25, 50 and 100 mg/kg/ day, i.p.	GD 14 - GD 19 (F1 generation) F1 generation was crossed among themselves to produce the F2 generation mice	100-days (F1 generation) 100-days (F2 generation) (No sex specification)	Halder et al. (158)
Sprague-Dawley rats	1 mg Cd/kg and 5 mg Cd/kg, corresponding to 1/90 and 1/18 of LD 50, respectively, oral route by gavage	GD 0 - PND 21	PND 35 PND 56	Feng et al. (159)
Wistar rats	0.3 mg Cd/kg and 0.6 mg Cd/kg, daily injected subcutaneously	GD 7 - GD 15	PND 45	Gumilar et al. (160)

Cd, cadmium; GD, gestational days; PND, postnatal days. *Male and female separated or as described in the line.

Couto-Moraes et al. (157) tested whether immediate postpartum testosterone administration (50 μL of testosterone 0.2% i.p.) could reverse the toxic effects of Cd in a model in which dams received 10 or 20 mg Cd/kg by gavage from GD 18–21 until the 7th lactation day. Signs of maternal toxicity were identified in the 20 mg Cd/kg group, including changes in weight, weight gain, and food and water consumption. Male offspring exhibited delayed physical and reflex development, particularly in the 20 mg Cd/kg group, which was not reversed by testosterone administration. Despite impairments in palmar grasp and negative geotaxis, locomotor and exploratory abilities in the Open-Field Test at PNDs 21 and 75 were unaffected.

Male offspring from dams exposed to 10 mg Cd/L in drinking water during gestation or lactation showed learning impairments in young adulthood (7 weeks old), detected by the Morris Water Maze Test, while female offspring displayed similar deficits in adulthood (12 weeks old) in the Y-Maze Test. These learning deficits were associated with structural changes in the hippocampal CA1 pyramidal cell layer and altered protein expression of different subunits of GABA receptor subtype A subunits (GABAARα5 and GABAARδ). Female offspring also exhibited significantly decreased serum estradiol levels in young adulthood (161).

Feng et al. (159) treated female rats with 1 or 5 mg Cd/kg (1/90 and 1/18 of LD_50_) by gavage from GD 0 to PND 21 and reported cognitive deficits in male offspring at PNDs 35 and 56. These changes were linked to synapse and neurite destruction in the hippocampus mediated by actin regulator Coronin-1a (CORO1A) via the Ras-related C3 botulinum toxin substrate 1 (RAC1)-p21-activated kinase 1 (PAK1)-Glycoprotein M6a (GPM6A) signaling pathway, which regulates filopodia formation. Cd was detected at low levels in the cerebral cortex but not in the hippocampus (<0.001 mg/kg) at PND 21. Using the same dose and experimental period (1 or 5 mg Cd/kg from GD 0 to PND 21), Mai et al. (162) recently demonstrated that maternal Cd exposure disrupts the interaction between aquaporin 1 (AQP1) and Hyperpolarization-activated cyclic nucleotide-gated channel 1 (HCN1), promoting amyloid-beta pathology and Alzheimer-like cognitive decline in male offspring. Cd exposure resulted in the persistent down-regulation of HCN1 in the hippocampus, which obstructed AQP1-mediated clearance, resulting in the accumulation of the amyloid-beta protein.

Parenteral routes have also been used in Cd intergenerational studies (Minetti & Reale, 2006; 158, 160, 163). Sensorimotor development was analyzed in offspring (sex not specified) at PNDs 3–7 from dams that received 0.3 or 0.6 mg Cd/kg/day subcutaneously from GD 7 to GD 15. Significant impairments in the Righting Reflex and Cliff Aversion Tests were detected in the 0.6 mg/kg group. At PND 45/90, both male and female offspring from this group showed reduced anxiety-like behavior in the Elevated-Plus Maze Test. Gumilar et al. (160), using the same doses and route from GD 7 to GD 15, found that only male offspring exhibited learning and memory deficits in the Step-Down Inhibitory Avoidance and Radial Maze tests. Halder et al. (158, 163 treated dams with 1.2 mg Cd/kg/day intraperitoneally from GD 14 to GD 19 to generate the F1 generation, which was then crossed to produce an F2 generation. Behavioral analysis at PND 100 (sex not specified) showed that F1 offspring had learning and memory impairments, while F2 offspring only exhibited improved short-term memory when dams received co-treatment with quercetin (100 mg/kg/day). These behavior changes were associated with cerebral oxidative stress, characterized by increased MDA levels, decreased glutathione levels, and altered activities of catalase (CAT) and glutathione-S-transferase (GST) enzymes.

Investigations also reported Cd exposure was linked to epigenetic changes in human and experimental models (164–166) (**Figure 2**). Human studies indicate that prenatal Cd exposure alters DNA methylation patterns in placental and neonatal tissues, with sex-specific methylation changes at loci such as paternally expressed 3 (PEG3), maternally expressed 3 (MEG3), miRNA expression such as miR-509-3p and miR-193-5p expression, and other regulatory regions that may impact genes involved in neurodevelopment and neuronal function. Although direct human cognition studies are limited, these epigenetic associations provide plausible molecular links between early Cd exposure and subsequent learning/memory outcomes (164, 166). Cd exposure has been associated with epigenetic dysregulation of non-coding RNAs (e.g., lncRNAs) that modulate neuronal mitochondrial function, cytoskeletal dynamics, and synaptic maintenance, contributing to persistent memory deficits. For example, chronic low-dose Cd exposure in mice upregulated the lncRNA *Gm10532* (lnc-Gm10532), which recruited the m6A methyltransferase methyltransferase-like 14 (METTL14) to enhance m6A modification of Fission 1 (Fis1) mRNA, increasing mitochondrial fission and impairing neuronal function—mechanistically linking epigenetic modification to memory impairment. These findings suggest that Cd influences learning and memory by dysregulating epitranscriptomic and chromatin-related mechanisms in the developing brain (165).

Thus, offspring exposed to Cd during gestation or lactation generally showed limited alterations in basic motor, exploratory, or social behaviors, but several studies revealed subtle neuroendocrine and cognitive vulnerabilities. Early work reported no major behavioral deficits at low Cd levels, although female offspring displayed an inverse association between RC3 gene expression and locomotor activity, along with sex-specific disruptions in ERα, ERβ, and PgR mRNA levels. Higher-dose exposures produced maternal toxicity and delayed physical and reflex development in male offspring, which were not reversed by postpartum testosterone treatment, even though locomotor behavior in young and adult animals remained largely intact. More recent findings demonstrated clear sex- and age-dependent learning impairments linked to hippocampal structural damage, altered neuronal marker signaling pathway. Studies using parenteral exposure routes further showed sensorimotor deficits, reduced anxiety-like behavior, and male-specific learning and memory impairments across developmental stages.

## 3 Future perspectives

Although concerns about the neuroendocrine and cognitive impacts of heavy metals as EDCs have grown substantially, research addressing their intergenerational consequences—particularly for MeHg, Pb, and Cd—remains strikingly limited. Robust, multigenerational datasets are urgently needed to more accurately characterize long-term risks for present and future populations, especially when integrated with biomonitoring programs and surveillance of contaminants in food and drinking water. A major constraint in current experimental designs is the frequent use of exposure levels that do not mirror concentrations found in human biological samples over time, thereby limiting translational relevance. In addition, most studies have centered almost exclusively on maternal transmission mechanisms, while potential effects transmitted through the paternal lineage remain comparatively understudied. Even fewer investigations assess both parental lineages simultaneously and model chronic, low-dose exposures that more closely reflect real-world human conditions.

Recent studies have examined associations between parental exposure to metal mixtures and adverse developmental outcomes in children, including increased risks of preterm birth, congenital anomalies, and abnormal birth weight (149, 167–170). Combined exposure to metal mixtures revealed that As levels in paternal toenails and Cd concentrations in maternal toenails were positively correlated with an increased risk of delayed neurodevelopment in offspring (168). Prenatal paternal Cd exposure was associated with reduced birth weight, particularly among female infants (167). Furthermore, paternal urinary concentrations of titanium (Ti), vanadium (V), chromium (Cr), manganese (Mn), cobalt (Co), nickel (Ni), copper (Cu), and selenium (Se), as well as maternal urinary concentrations of V, Cr, Ni, Cu, Sb, and antimony (Sb), were associated with a 21- 91% increased risk of birth defects (169). In addition, paternal and maternal urinary Sb levels were associated with a 45% and 43% higher risk of preterm birth, respectively (170).

Real-life scenarios typically involve exposure to complex mixtures of EDCs—including phthalates, bisphenols, per- and polyfluoroalkyl substances (PFAS), polycyclic aromatic hydrocarbons (PAHs), microplastics, and multiple heavy metals—yet human and experimental work examining combined or interactive effects on neurodevelopment, behavior, and memory is still scarce (171–173,174–176). This gap is particularly concerning given that, in modern society, humans encounter chemicals continuously through food, water, air, consumer products, and cosmetics, via inhalation, dermal absorption, ingestion, and, less commonly, injection. As such, we are effectively living in a chemically pervasive environment, and many questions regarding long-term neuroendocrine and cognitive outcomes remain insufficiently addressed.

Recent human and experimental evidence has demonstrated a clear association between exposure to mixtures of heavy metals and adverse cognitive and mental health outcomes (Dewey & Soomro, 2025; 174, 175; Chandra et al., 2026). There is strong support for the contention that elevated prenatal and early-childhood exposure to As, Cd, Pb, Hg, and their mixtures is associated with impaired cognitive, motor, and behavioral development, as well as increased risk of mental health disorders in children (Dewey & Soomro, 2025). It was recently demonstrated that premature brain aging in rats is induced by chronic low-level exposure to a mixture of Pb (10 mg/L), Hg (0.05 mg/L), and Cd (3.5 mg/L) via drinking water from mating until offspring weaning. Then, the offspring were exposed to the mixture (3.5 mg/L Pb, 0.015 mg/L Hg, and 0.5 mg/L Cd) for 32 weeks. This exposure protocol induced cognitive impairment, specifically affecting spatial memory and social cognition by promoting hippocampal telomere dysfunction, neuronal loss, and structural synaptic plasticity damage (CA1/CA3 regions) (175).

Similarly, Chandra et al. (2026) reported that perinatal exposure to a metal mixture in drinking water- comprising Pb (15 ppb), As (10 ppb), Cd (5 ppb), and Cr (VI) (100 ppb)- in female mice increased anxiety-like behavior and impaired short-term memory, without affecting working memory. At the cellular level, pyramidal neurons in the medial prefrontal cortex (mPFC) and hippocampal Cornu Ammonis 1 (CA1) region exhibited increased intrinsic excitability. In addition, mPFC neurons showed enhanced amplitudes of spontaneous excitatory postsynaptic currents, ultimately leading to disrupted neuronal function and altered behavior. Gestational exposure to Cd (0.3 mg/kg/day) and to the PAH benzo[a]pyrene (BaP, 0.03 mg/kg/day), either alone or in combination, was related to lasting neurobehavioral and developmental impairments in rat offspring that persist into adulthood (174).

Other important aspect is sex specific changes in memory and learning after these metals’ exposure (135,177, 178). While epidemiological evidence suggests differential susceptibility by sex, with experimental studies indicating changes neurochemical signaling in males following prenatal metal exposure, the complete mechanistic sex differences remain underexplored (179, 180). These prenatal and early postnatal exposure my reflect sex differences in metal toxicokinetic, hormone-regulated detoxification, nuclear and membrane receptors, oxidative stress pathways, and neural epigenetic control, suggesting that endocrine modulation could underlie sex-biased neurobehavioral outcomes from these metals’ exposure (178–180).

## 4 Conclusions

Perinatal exposure to MeHg, Pb and Cd clearly emerges as a critical window of vulnerability for long-lasting neurobehavioral disturbances, with converging evidence across models demonstrating persistent impairments in motor function, learning, memory, emotional regulation, and social behavior. Mechanistically, oxidative stress, excitotoxicity, neuroinflammation, disrupted neurotransmitter systems, and altered synaptic plasticity appear to be shared pathways, while disruptions of neuroendocrine signaling- including thyroid, adrenal, gonadal, and stress-axis pathways- likely modulate the behavioral phenotypes. Despite substantial evidence for early-life vulnerability, major gaps remain regarding dose-response relationships, the neuroendocrine mechanisms governing sex-dependent sensitivity, and the long-term consequences of low-dose exposures that reflect contemporary human environmental conditions.

Regardless of the effects of their endocrine-disrupting properties, heavy metals can directly affect synaptic plasticity by crossing the blood-brain barrier, accumulating in specific brain regions, such as the hippocampus, and inducing neurotoxicity (20, 22). Evidence beyond correlation confirms that heavy metals (specifically Me-Hg, Pb, and Cd) are related to cognitive deficits by acting as EDCs that directly interfere with hormone synthesis, transport, and receptor signaling, compromising neural plasticity and neurodevelopment (28). Thyroid hormone disruption and steroid receptor interactions are two causal mechanisms that relate heavy metal-induced endocrine disruption to cognitive deficits (11, 150, 153, 154). Additional mechanisms involved the disruption of immunomodulatory glucocorticoid effects and the HPA axis (56, 80, 82, 84). Pb interferes with neurotransmitter synthesis and storage by mimicking essential calcium (Ca²^+^), inhibiting N-methyl-d-aspartate (NMDA) receptors, which are critical for synaptic plasticity, learning, and memory (126, 127). In animal models, the presence of these heavy metals results in oxidative stress, inflammation, and cell death in neural tissues, which are specifically correlated with impairments in hippocampal function (66, 73, 117, 128, 161; Chandra et al., 2026). Furthermore, these mechanisms demonstrate that the cognitive decline is not solely related to the presence of heavy metals but is also directly caused by their disruption of the endocrine system’s regulation of brain function.

Future research must prioritize integrative approaches that combine developmental neurotoxicology with endocrine, epigenetic, and circuit-level analyses to clarify how MeHg, Pb, and Cd exposure disrupt neurodevelopmental trajectories (137). Considering the growing recognition that early-life exposure to EDCs shapes disease risk across the lifespan, next-generation studies should incorporate multigenerational and transgenerational designs, assessments of maternal behavior, and the influence of environmental modifiers such as nutrition, stress, and enrichment. Advanced imaging, single-cell omics, and functional neuroendocrine profiling will be essential for identifying biomarkers of early disruption and predicting long-term outcomes. Ultimately, translating mechanistic insights into public health strategies requires establishing human-relevant exposure thresholds, improving biomonitoring tools, and recognizing that even low-level exposures during sensitive developmental windows may produce subtle but biologically meaningful effects that manifest only in adolescence or adulthood.
